# ReOpen demands as public health threat: a sociotechnical framework for understanding the stickiness of misinformation

**DOI:** 10.1007/s10588-021-09339-8

**Published:** 2021-08-10

**Authors:** Francesca Bolla Tripodi

**Affiliations:** 1 School of Information and Library Science, University of North Carolina, Chapel Hill, USA; 2 Center for Information Technology and Public Life, University of North Carolina, Chapel Hill, USA

**Keywords:** ReOpen, COVID-19, Misinformation

## Abstract

In the absence of a national, coordinated, response to COVID-19, state and local representatives had to create and enforce individualized plans to protect their constituents. Alongside the challenge of trying to curb the virus, public health officials also had to contend with the spread of false information. This problematic content often contradicted safeguards, like masks, while promoting unverified and potentially lethal treatments. One of the most active groups denying the threat of COVID is The Reopen the States Movement. By combining qualitative content analysis with ethnographic observations of public ReOpen groups on Facebook, this paper provides a better understanding of the central narratives circulating among ReOpen members and the information they relied on to support their arguments. Grounded in notions of individualism and self-inquiry, members sought to reinterpret datasets to downplay the threat of COVID and suggest public safety workarounds. When the platform tried to flag problematic content, lack of institutional trust had members doubting the validity of the fact-checkers, highlight the tight connection between misinformation and epistemology.

## Introduction

In the absence of a coordinated, national response to the COVID pandemic, many state and local officials had to create and enforce their own measures to protect their constituents. In addition to curbing the spread of the virus, public health officials had to also contend with the similarly rampant and dangerous spread of false information. This problematic content often contradicted, discouraged, and protested public health precautions while promoting unverified and potentially lethal treatments.

Some of the most active coronavirus skeptics were part of The Reopen the States Movement (aka ReOpen). Despite the mounting toll of COVID diagnoses and deaths in the United States, group members pushed for schools, businesses, and places of worship to reopen. Groups first began to form on Facebook in response to the closure of churches over Easter Sunday (April 12, 2020). However, the disinformation activity that fuels ReOpen is not exclusive to any one digital platform, or even to online media in general.

Given the potential risk ReOpen groups pose to the public, this paper argues for a multidisciplinary approach to understanding the effects of media manipulation as it pertains to COVID. While big data is an important mechanism for understanding false information as it pertains to the COVID pandemic, this study underlines why researchers must combine data-driven analysis with a sociotechnical model of audience ethnography (Marwick 2018) to better understand how false narratives are created, believed, and shared, as well as how it might be combatted.

Drawing on qualitative data, I find that central narratives shared in ReOpen groups are connected to the “deep stories” of American conservatism (Hochschild 2018; Polletta and Callahan 2017). These conclusions mirror recent research by scholars at MIT who found that people manipulate COVID data to reflect their existing logics and sociopolitical positions (Lee et al. 2021). By observing public ReOpen groups on Facebook, this study further demonstrates how health misinformation drives political action.

## Theoretical background

The ability to access and analyze large datasets has transformed social scientific research. Data science helps researchers understand actions, behaviors, and choices within networked publics over short periods of time and provides the opportunity to understand misinformation ecosystems, detect bot-driven activities, and track social movements and activism within and across platforms (Benkler et al. 2018; Freelon et al. 2016; Freelon et al. 2020; Jackson et al. 2020; Lazer et al. 2020; Singh et al. 2020; Tacchini et al. 2017; Uyheng and Carley 2020).

At a conference hosted by Carnegie Mellon University’s Center for Informed Democracy and Social-cybersecurity (IDeaS), panelists shared their emergent findings on how the spread of misinformation impacts human activity during periods of social upheaval. Researchers of Twitter and YouTube content and interactions found that extreme polarization and hyper-partisanship increases during times of crises (e.g., a global pandemic), and explained that when false narratives move across platforms, it strengthens their perceived legitimacy (Kirdemire and Agarwal 2020). Analysis of 200 million COVID-related tweets revealed that hate-speech surrounding the pandemic shifted over time. While xenophobic hate speech drove early discussions surrounding COVID (e.g., “the China virus,”), Twitter users then hashtagged those phrases to call out bigoted political leaders stoking hate (Uyheng et al. 2020).

However, researchers’ ability to gain access to “raw data” is often incompatible with the privacy and intellectual property concerns of the platforms, making reproducibility and replication difficult (Lazer et al. 2020). More importantly, the in-house researchers who make datasets publicly available do so in the interests of the institutions for which they work and the platforms those institutions represent, which affects how social scientists can analyze “raw” data (Anderson 2020; boyd and Crawford 2011; Marres 2018; Roose 2021, https://www.nytimes.com/2021/07/14/technology/facebook-data.html). Since its inherent focus is traceable behavior and visual metrics (e.g., hashtags, shares, follows, and likes), big data can also miss out on the “sticky stuff” (Wang 2016): the meaning-making processes behind why users relate to and believe false information, how they share those interpretations with social media networks, and the ways value systems are integrated into information appeals (Baym 2013).

Misinformation researchers have explained that the extent to which audiences believe and share problematic content is connected to the institutional and epistemological nature of knowledge production (Anderson 2020; Benkler et al. 2018; Lee et al. 2021; Marwick 2018; Tripodi 2018). To understand how cultural practices and technological affordance both constrain and enable certain meanings and actions, ethnographic work is essential (Marwick 2018). Contextualizing the ideological appeals behind alternative facts gives researchers an interactional view of how communities leverage the language of science and media literacy to spread misinformation (Lee et al. 2021). Qualitative research allows scholars to consider the sociotechnical aspects of media manipulation.

The earliest studies of sociotechnical theory came long before the internet. In the late 1940s/early 1950s, Trist and Bamforth studied coal mining in England and found that mechanization had decreased productivity because the technology did not consider the human/social aspects necessary for job performance. They argued that for organizations to function well, they need to optimize both technical processes and the social systems operating within them (Trist and Bamforth 1951). By recognizing the interdependence between social and technical systems, Bijker (1995) highlights that both “technology and society are human constructs” (p. 3) and stressed the importance of linking “micro stories with macro structures” (p. 4). Qualitative studies on how groups use and interact with information on Facebook sheds light on the context behind why misinformation is believable (Marwick 2018). Media manipulation is more than simply a bug in the code; it is a *sociotechnical vulnerability*.

## Methods

Given ReOpen’s initial response to the closure of churches on Easter Sunday that emphasized “freedom and faith,” I sought answers to the following research questions:

1. Do ReOpen members align with political positions/rhetoric besides COVID mandates?
2. Does the information shared within these groups align with the affective messaging of a polarized media ecosystem?
3. In what ways do these groups’ calls to action directly contrast warnings from public health officials and how might this pose a health-security threat in the United States?

This paper relies on a sociotechnical model of media effects (Marwick 2018) to analyze how ReOpen actors, messages, and affordances influence the spread of problematic information surrounding COVID. To do so, I engaged in “deep lurking” (Lee et al. 2021) of *public* ReOpen community groups on Facebook situated in four states across the United States (e.g., ReOpen Florida). To maintain confidentiality, specific states are withheld in publication, but one state from the East, the West, the South, and the North of the United States was selected to provide a wide range of political leanings and geographic locations. By employing a case study approach (Small 2009) to studying the ReOpen movement and reducing the data points to allow for hand-coding and depth (Latzko-Toth et al. 2018) this study provides a holistic understanding of ReOpen culture and what information they trust.

I conducted these observations through a research-specific Facebook account from April 12, 2020 to December 12, 2020. Observations consisted of daily visits to the four ReOpen groups, tracking themes, taking screenshots, and noting information being used to support group members’ arguments as they were discussed in threads (i.e., posts, links, memes, events, comments, likes or other reactions). This digital ethnographic approach is grounded in the assumption that on and off-line behavior is increasingly interconnected (Baym 1998) and provides a situated understanding of the meaning-making processes ReOpen members engage in when trying to make sense of the COVID pandemic.

In addition to ethnographic observations, I conducted a qualitative analysis of the content shared in the groups (e.g., news stories, comments, memes). Rather than focus on the frequency of phrases, I sought to identify the underlying patterns and processes of ReOpen culture (Altheide 2000). This method provided a systematic yet flexible way to collect and analyze data simultaneously (Charmaz and Thornberg 2020). As a method, “grounded theory” allows researchers to move beyond description and construct new concepts that explicate what is happening (Charmaz 2006). As such, my findings generate meaningful insights regarding the connections between ReOpen mantras and right-wing media information systems, but does not imply generalizability nor lead to immediate, quantifiable results.

## Findings

During my observations, a central theme emerged among members doubting the severity of the virus. Those pushing to reopen churches, businesses, and schools did so because they did not inherently trust the information about COVID provided by mainstream media or their state government, especially when local policies contradicted President Trump’s assessment of the virus. Because members did not trust government or mainstream media, they sought alternative sources who interpreted data about COVID to downplay the threat, flout public safety measures, and promote workarounds. Lack of trust in “the media” also spilled over onto the platform such that members seemed to believe problematic information more when it was flagged by Facebook as false or misleading.

### Downplaying the threat

A central tagline circulated in the ReOpen groups I observed was that “the cure is worse than the disease.” This motto hinges on the logic that COVID is just like the flu and because states don’t usually close schools and businesses down during flu season, they shouldn’t do it now. A central component of this argument is tied to denying COVID’s disproportionate fatality statistics while simultaneously circulating alternative interpretations of available datasets. Through this analysis, ReOpen members believe that closing schools, places of worship, and businesses are more disastrous than the disease, because COVID mortality is lower than reported.

Over the course of eight months, ReOpen members repeatedly cited unverifiable information from non-credible sources (e.g., personal blogs or vlogs with no medical credentials) to support this claim. These personal posts often featured the authors’ own interpretation of data to prove that “the media” and “elites” were inflating hospital capacity numbers at the expense of working-class jobs. Such use of “counter-visualizations” suggests that ReOpen members are similar to “anti-maskers” in their prolific use of creating and sharing data visualization models to spread misinformation that the pandemic is exaggerated (Lee et al. 2021). By drawing on “the data” it allowed members to make more outlandish and racist conspiracies surrounding George Soros, Bill Gates, and undocumented migrants seem more plausible while appealing to a core conservative value that government institutions are not trustworthy (Peck 2018).

For example, ReOpen members across groups shared a November 2020 blog post claiming that Bill Gates inflated COVID fatality figures to ensure that American schools stayed online, and that “Mexicans” were being transported by the firetruck-load to “jam El Paso hospitals.” In this blog post “Helena” conducted her own analysis of publicly available hospital data in order to “prove” the numbers “didn’t add up.”

Members also shared the *Gateway Pundit* story claiming that the CDC “quietly” changed COVID death rates, and that the actual numbers were “more typical to a seasonal flu.” Group members supported these claims by relying on the hermeneutical method of *scriptural inference,* a distinctly conservative media literacy practice that leverages literal translation of texts over professional interpretation of documents (Tripodi 2018). For example, members often quoted an NPR report that stated people die “*with* COVID” not necessarily *“from* COVID.” Sometimes, members posted CDC hyperlinks alongside their own calculations to make it seem like the unsubstantiated claim that “only 6% of all coronavirus deaths were completely due to the coronavirus alone” was backed by “the data.” Individualized and unqualified interpretations of data empowered ReOpen members into thinking that they knew the truth about COVID, while those following guidelines were the ones being tricked.

According to recent investigative journalism, some health departments have indeed been manipulating COVID data; the investigations find, however, that data manipulators lied to reduce positivity rates in order to facilitate and actualize ReOpen movement goals (Wamsley 2020). During my eight months of ethnographic observations, ReOpen Facebook groups never discussed data manipulation efforts that supported ReOpen measures.

### We Will Not Comply!

Conceptual frames and previous experiences guide human responses to new situations (Goffman 1959). Persistent patterns of cognition and interpretations are key to understanding how ReOpen groups organized information they trusted regarding the pandemic (Gitlin 1980). Since ReOpen members believed the casualties of COVID were inflated by medical and media elites, they subsequently framed social-distancing and mask-wearing as unforgivable deprivations of freedom. Memes, videos, and news media group members shared supported these assertions, arguing that COVID was a guise to test the levels of control the government could exercise without mass protest.

**Fig. 1:**
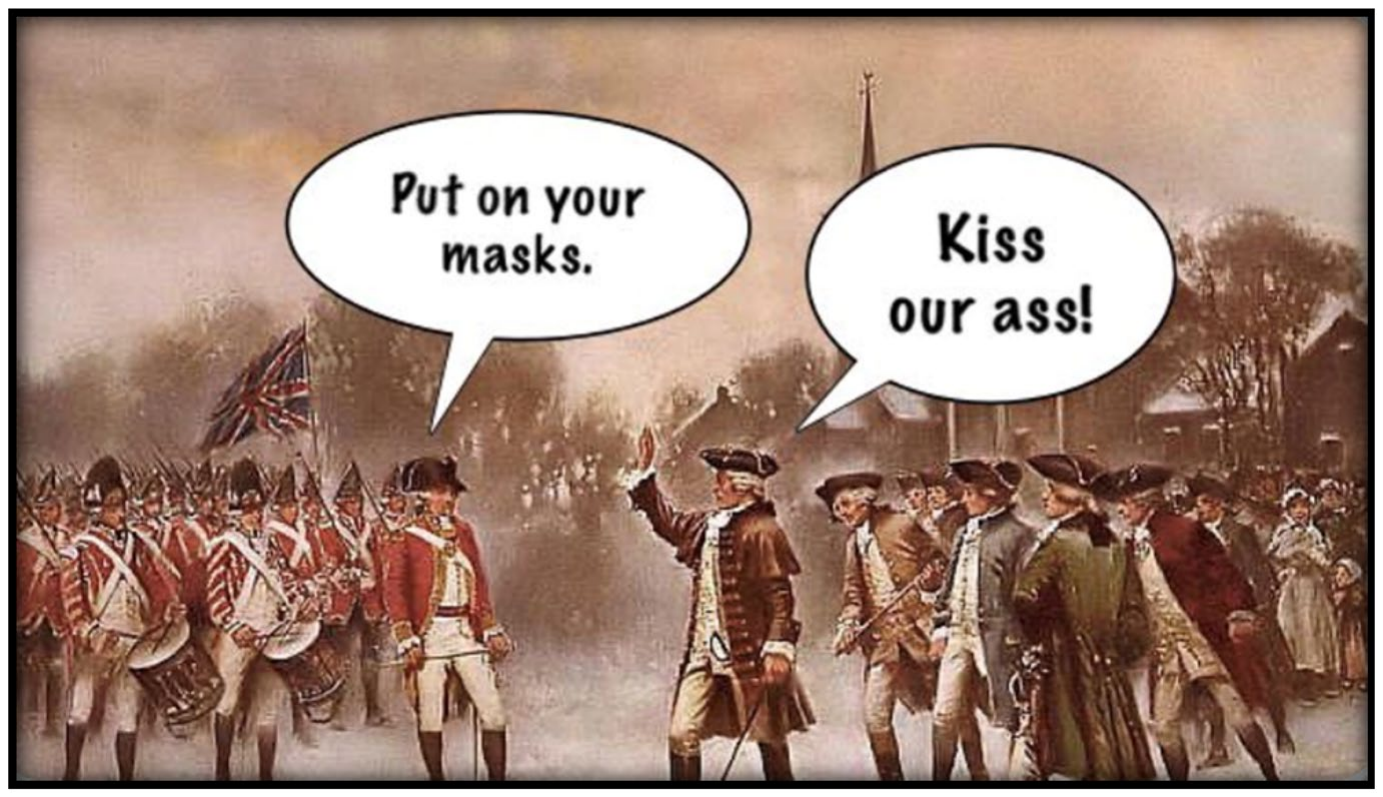
A meme shared in October 2020 equating the ReOpen movement with colonial revolutionaries

Many states that imposed social-distancing and mask-wearing mandates were Democrat-run. ReOpen groups galvanized around this lopsided decision making, referring to masks and social distancing measures as “socialist” agenda setting. These arguments were often layered with conspiratorial accusations that masks were simply a way for the “radical left” to control the public. As the 2020 Presidential election drew closer, ReOpen members urged others to “vote for freedom,” drawing analogies between ReOpen and the colonial revolutionaries in North America (see Fig. 1).

ReOpen group members framed themselves as patriots fighting against a repressive government, while people complying with regulations were referred to as sheep or “sheeple.” Content shared in groups and at rallies made frequent analogies between public health measures and both slavery and Nazi Germany. ReOpen members across groups posted comments that they “weren’t a slave” to the government or that the purported inflation of COVID cases was being used by the government to “keep them scared, keep them enslaved!” They also argued that measures being enacted were not unlike those used by Adolf Hitler (e.g., a caption reading “governor declares new fascist COVID rules”).

A similar sentiment was captured by reporters at a May 16, 2020 anti-lockdown rally protest where a woman held a photograph of Escrava Anastacia, an enslaved African woman forced to wear an iron mask alongside the statement “MUZZLES ARE FOR DOGS AND SLAVES. I AM A FREE HUMAN BEING.” In April 2020, an Idaho States representative made a contrast between public health measures and Nazi Germany: “I mean that’s no different than the Nazi Germany, where you had government telling people, you are an essential worker or non-essential worker and the non-essential workers got put on a train” (Kesslen 2020). Representative Green (R-Ga.) compared mask-wearing requirements at the Capitol to the Holocaust and insinuated that government officials encouraging vaccination were akin to Nazi soldiers (Shabad et al. 2021; Shabad 2021).

In response to mask mandates, the catchphrase #WeWillNotComply began circulating throughout ReOpen groups. The historic nature of this hashtag is also rooted in conservative ideals. The call for non-compliance originated at a “Second Amendment Sanctuary” rally protesting gun-control bills recently introduced in the Virginia state legislature (Swearer 2020). Associating social-distancing and mask-wearing as violations of individual freedoms further frames the movement as a conservative effort.

By framing resistance as a call to freedom and compliance being a form of (forced) submission, #WeWillNotComply served a battle cry for ReOpen. It allowed members to proudly assert their positions in public by refusing to wear a mask and positioned people obeying public safety measures as “sheeples” that had been tricked/fooled into thinking COVID was real because they did not “do their own research” on the subject.

### Promoting workarounds

**Fig. 2:**
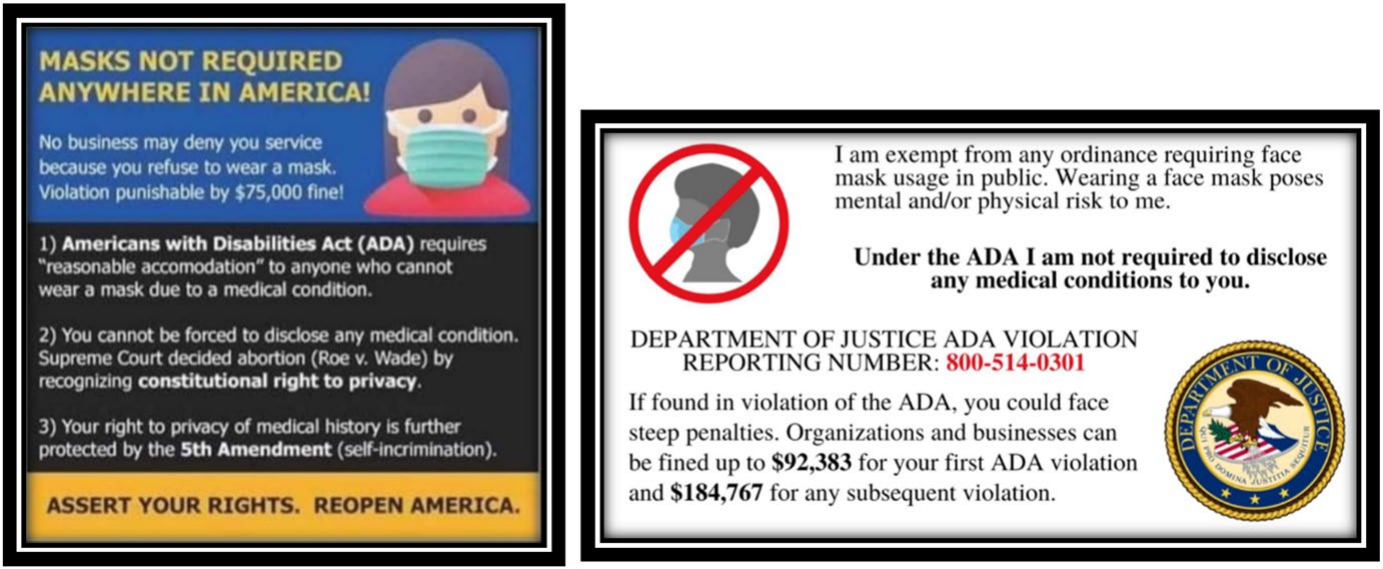
Pamphlets shared on ReOpen groups encouraging members to claim a medical exemption, so they do not have to wear a mask in public—Posted May 2020

ReOpen members first began circulating information in May 2020 that mask-wearing mandates were a civil rights violation punishable by a $75,000 fine. Applying the practice of scriptural inference to the Americans with Disabilities Act (ADA), the 5th Amendment to the Constitution, and the 1974 Supreme Court decision Roe v. Wade (410 U.S. 113), ReOpen members posted pamphlets asserting their individual rights and encouraged members to print and carry them when they went out in public (see Fig. 2). These pamphlets drew out cherry-picked phrases from long, complex, legal texts to help ReOpen members lie about having a medical condition in order to avail themselves of mask-wearing mandates. This two-step process of claiming a medical condition and then referencing a government protected right to privacy was captured in a viral video a grocery store customer filmed herself when trying to enter the facility without a mask (McAboy 2020).

Accompanying the pamphlets were tips for how to avoid wearing a mask in public. In one group, a mom shared that she bought masks for her kids that tie in the back and suggested parents only tie the top one or tie the bottom one loosely. Doing this allows for her kids to “pretend like they are playing the game” while doing their own thing. This was also a strategy on election day, when posts in each of the groups I was observing noted which polls were not monitoring mask coverage and encouraged members to vote without a mask. Members frequently suggested people just carry a mask in their hand, pocket or purse, but not actually put it on their face unless a clerk “ask” or “try to force” them to do so. Doing this allows them to enter stores freely but put the pressure on low-wage workers (or volunteer poll workers) to enforce the rules.

They also shared strategies to try and fool public health data used to guide school closing decisions. Termed the “mom code,” this tactic encouraged parents to refuse to test their children if they came down with COVID symptoms to reduce the number of COVID cases in their district.

During this project, my dedicated research account was targeted with curated advertising centered around sidestepping mask mandates. On November 13, 2020, at 11:47 am, I noticed an advertisement for “The Fake Mask.” Clicking on the link took me to fakemaskusa.com, a company started by “a few dudes from Ohio” that just want to “help people breathe better.” The company sells an entirely mesh and fully porous mask with the following product description: “Guess what? Cloth masks don’t do anything. Neither does this masterpiece. But as least you can breath (sic) now. The Fake Face Mask is the most breathable, comfortable face covering on the market.”

### Distrust in the “fact-checking” process

Often fact-checking efforts are complicated by the context specificity of posts (Roberts 2019). This was increasingly difficult given the US government’s failure to communicate consistent messaging surrounding COVID-19 (Balog-Way and McComas 2020). Mixed messages regarding mask wearing not only complicated governments ability to protect its citizens but also eroded trust in self-protection measures and made it increasingly challenging for the public to distinguish between scientific evidence and misinformation (Collins et al. 2020; Sauer et al. 2021).

ReOpen members capitalized on this public-health confusion by posting links or clips of Dr. Fauci’s March 8th interview on *60 Minutes * to support their claims that masks should not be required in public. These posts, as well as the original video, are still available both on Facebook and YouTube. While these posts are accompanied with a disclaimer urging viewers to “Get the latest information from the CDC about COVID,” the flag did little to prevent ReOpen members from using the clip out of context. This finding further complicates media literacy methods aimed at combatting the spread of problematic content. When ReOpen members 2018 lift and misinterpret the direct words of Dr. Fauci, it makes it almost impossible to automate content moderation decisions.

Such ambiguities fuel ReOpen members’ beliefs that the fact-checking process is biased against conservatism. This distrust of fact-checking was evident in the disdainful and scornful language ReOpen members used to cite their sources (e.g., “News source for our big brother ‘fact checkers’”). Posters would frequently use scare-quotes around the phrase “fact-checking” to indicate doubt in the information verification process. On multiple occasions the presence of a fact-check only served to *further validate* the credibility of the source or claim. For example, in one group a user posted a link to a story in *The Federalist* arguing that only “science-deniers” believe in mask mandates. Facebook flagged the content as “Partly False Information.” However, comments under the article claimed that since Facebook fact checkers deemed it false, “you know it’s true,” and further validated members’ resolve that masks were an unnecessary precaution.

When the platform removed false information, ReOpen members did not discuss the merits of the original information, but instead insisted that Facebook was purposefully suppressing conservatism. For example, in post from October 2020 that was subsequently removed, ReOpen members vented their frustrations in the comments below the notice that the link was no longer visible. In the thread, one member argued that “doctors and nurses” should decide what information is available, not Facebook, and another lamented that the platform was turning into “the thought police!” Other members boasted about how often they were put in Facebook or Twitter “jail,” joking that they should create badges for members who are repeatedly kicked off for trying to spread “the truth.”

**Fig. 3:**
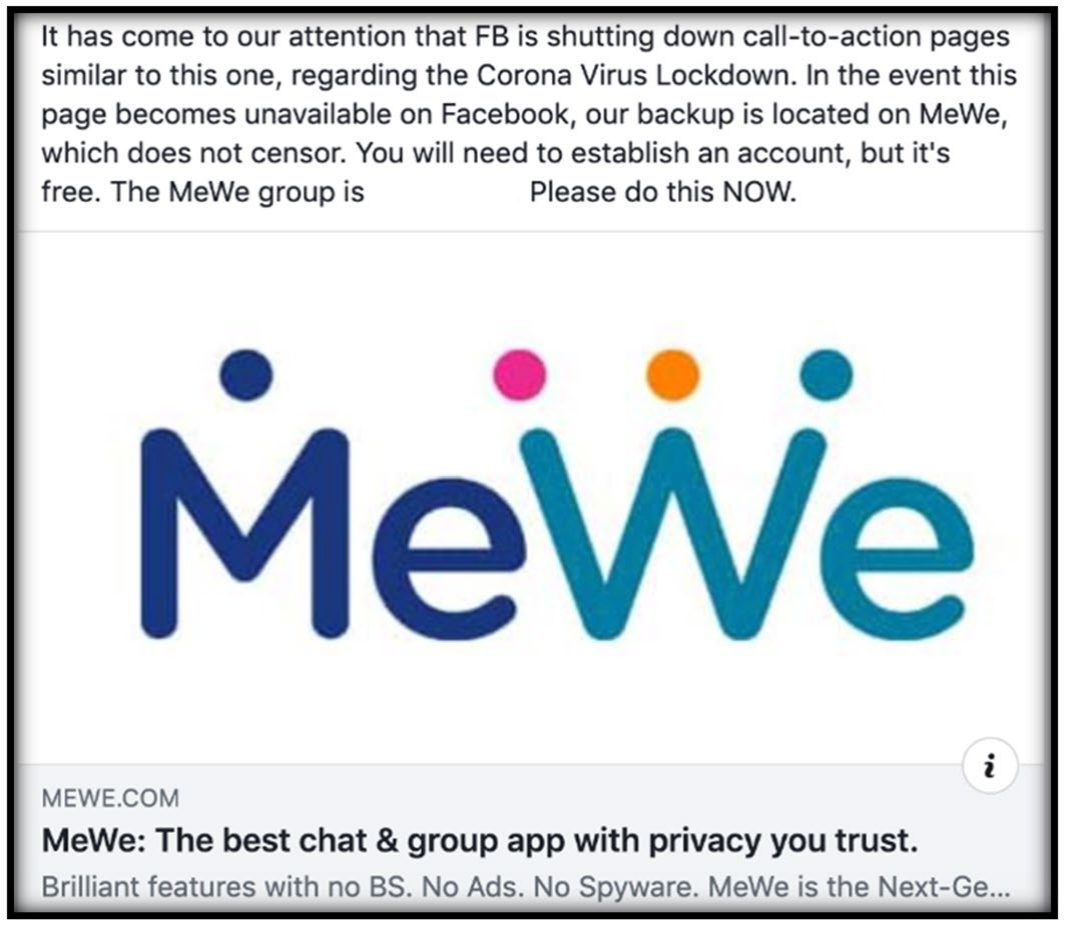
ReOpen post with MeWe information should Facebook ReOpen Group be removed

Like the workarounds for wearing masks, users would also share technical loopholes to keep false information accessible. Early on ReOpen groups thought they would be banned by Facebook. To ensure a potential Facebook ban wouldn’t hurt the movement, ReOpen members set up groups on alternative platforms just in case, but used Facebook to circulate information about these digital safehouses (Fig. 3).

In July 2020, Facebook took down a 45-min livestream video of Breitbart’s that featured doctors promoting hydroxychloroquine as an effective treatment for preventing COVID. Calling itself the “White Coat Summit,” the doctors argued that the public did not need to wear masks or socially distance when we had a cure (i.e., hydroxychloroquine). After ReOpen members posted that they could no longer access the video, others instructed them to “just got to Breitbart dot com to see the video.”

### Off-line organization

Not only were these groups tools for sharing information online, they were also tools for organizing action off-line. In June 2020, ReOpen groups in multiple states organized protests called “No Mask Saturday” with the goal of ending mask orders nation-wide. In November 2020, groups used the same strategies to defy a new round of curfews and stay-at-home orders intended to curb a spike in COVID cases. After the Presidential election, ReOpen groups quickly galvanized around President Trump’s “stop the steal” mantra, organizing a series of rallies devoted to what they perceived to be a stolen election. ReOpen members also organized events to gather signatures to prevent school closures or to try and recall governors in*-*person. Despite the public health risks associated with large gatherings, it is telling that ReOpen members opted for collecting hand signatures rather than circulating an on-line petition.

Sites for in-person ReOpen rallies were indicative of ReOpen’s connection to conservative values; Multiple events were scheduled in Hobby Lobby parking lots, for example. Hobby Lobby holds cultural significance to conservatives after the CEO of the organization took a public stance against the Affordable Care Act for mandating that companies provide access to contraception and the morning-after pill (Green 2013).

ReOpen groups were as skeptical of vaccines as they were of the virus that necessitated their development. In the early fall of 2020, ReOpen members urged others to skip the annual flu vaccine, falsely claiming that it made you more susceptible to catching COVID. In recent months, the groups have shifted focus to newly discovered, approved, and released COVID vaccines. Vaccines became the center of a new slippery slope narrative in which members warned that social-distancing and mask-wearing mandates would soon lead to vaccine mandates. They warned that Biden would impose vaccinations to go back to school and again drew on Holocaust tropes (e.g., those who didn’t vaccinate would be marked with yellow bans). Since ReOpen members still doubted the severity of the disease and believed it was being used by the government to control its citizens, many posted that they would not get the vaccine even if it prevented their kids from going back to school.

## Conclusion

By providing an in-depth analysis of the topics and information shared in four public ReOpen Facebook groups across the United States, this paper provides a textured understanding of how and why problematic content is believed and shared.

Ethnographic observations reveal parallels between ReOpen principles and conservative values in the United States more broadly (e.g., freedom and distrust in media institutions). Since members did not trust mainstream media, government, or public health reporting on the COVID pandemic, many returned to the decidedly hermeneutical and conservative scriptural inference process to “do their own research” on the numbers and then share their own individualized, unverified, and often contradictory interpretations of COVID data with their ideological social networks.

This proliferation of individualized, biased, and inexpert interpretations of complex data fueled another central idea: that the mainstream media, medical elites, and the radical left had overblown COVID to install a socialist regime and control the public. These same ideas were regularly supported by prominent members of the right-wing media (e.g., Tucker Carlson and Rush Limbaugh) and high-ranking members of the Republican party (including the outgoing President of the United States) who continuously downplayed the threat of COVID throughout the pandemic. Such logic both emboldened ReOpen efforts, but also framed those who complied with public health recommendations/requirements as the cultural dupes who had been fooled by the radical left.

Findings from this study could be bolstered with computational methods. For example, tracking the hashtag #WeWillNotComply on sites like Twitter or the presence of the phrase on YouTube videos can provide important insights into the reach of certain elements of misinformation (e.g., vaccine hesitation, denying mask efficacy or asymptomatic transmission). Using the Tubular Labs, a global video measurement and analytics platform, content surrounding the phrase “We Will Not Comply” promoted pro-gun, anti-mask, and COVID skepticism in their messaging. Public-health officials could use these same insights to help understand which geographic areas or demographic groups are less likely to support COVID vaccination efforts. Data scientists could also scrape and analyze comments associated with flagged or removed posts to see if other users are equally skeptical of platforms’ efforts to stop the spread of misinformation or if that is a perception exclusive to ReOpen members. Such analysis could provide important insights into the efficacy of fact-checking initiatives as well as explore alternatives to the problem of combatting misinformation on social media platforms.

## References

[CR1] Altheide DL (2000). Tracking discourse and qualitative document analysis. Poetics.

[CR2] Anderson CW (2020). Fake news is not a virus: on platforms and their effects. Commun Theory.

[CR3] Balog-Way DH, McComas KA (2020). COVID-19: Reflections on trust, tradeoffs. J Risk Res.

[CR4] Baym N, Jones S (1998). The emergence of on-line community. Cybersociety: communication and community.

[CR5] Baym N (2013) Data not seen: the uses and shortcomings of social media metrics. First Monday. Available at https://firstmonday.org/ojs/index.php/fm/article/view/4873/3752/. Accessed 10 Dec 2020

[CR6] Benkler Y, Faris R, Roberts H (2018). Network propaganda: manipulation, disinformation, and radicalization in American politics.

[CR7] Bijker WE (1995). Of bicycles, bakelites, and bulbs. Toward a theory of sociotechnical change.

[CR8] boyd D, Crawford K (2011) Six provocations for big data. In: A decade in internet time: symposium on the dynamics of the Internet and Society. Available at https://ssrn.com/abstract=1926431. Accessed 24 Dec 2020

[CR9] Charmaz K (2006). Constructing grounded theory: a practical guide through qualitative analysis.

[CR10] Charmaz K, Robert T (2020). The pursuit of quality in grounded theory. Qual Res Psychol.

[CR11] Collins A, Florin M-V, Renn O (2020). COVID-19 risk governance: divers, responses and lessons to be learned. J Risk Res.

[CR12] Freelon D, McIlwain C, Clark MD (2016) Beyond the hashtags: #Blacklivesmatter, #Ferguson, and the online struggle for offline justice Center for Media and Social Impact. Available at https://cmsimpact.org/blmreport. Accessed 12 Dec 2020

[CR13] Freelon D, Marwick A, Kreiss D (2020). False equivalencies: online activism from left to right. Science.

[CR14] Gitlin T (1980). The whole world is watching.

[CR15] Goffman E (1959). The presentation of self in everyday life.

[CR16] Green D (2013) Hobby Lobby CEO: here’s why Obamacare is a total affront to my religious beliefs. Business Insider. Available at https://www.businessinsider.com/hobby-lobby-ceo-david-greens-obamacare-statement-2013-9. Accessed 14 Dec 2020

[CR18] Hochschild AR (2018). Strangers in their own land.

[CR20] Jackson SJ, Bailey M, Welles BF (2020). #HashtagActivism.

[CR21] Kesslen B (2020) Idaho lawmaker under fire for comparing state coronavirus response to Nazi Germany. NBC News. Available at https://www.nbcnews.com/news/us-news/idaho-lawmaker-under-fire-comparing-state-coronavirus-response-nazi-germany-n1187651. Accessed 21 Apr 2020

[CR22] Kirdemire B, Nitin A (2020) Social Media, news, polarization, and disinformation in times of crisis: a case study on Turkey. In: Presented at the Social-Cybersecurity in Times of Crisis and Change IDeaS Virtual Institute—November 19–20, 2020

[CR23] Latzko-Toth G, Claudine B, Mlanie M (2018). Small data, thick data: thickening strategies for trace-based social media research. The SAGE handbook of social media research methods.

[CR24] Lazer DM, Pentland A (2020). Computational social science: obstacles and opportunities. Science.

[CR25] Lee C, Yang T, Inchoco G, Jones GM, Satyanarayan A (2021) Viral visualizations: how coronavirus skeptics use orthodox data practices to promote unorthodx science online. In: CHI Conference on human factors in computing systems CHI ’21), May 8–13, 2021, Yokohama, Japan. 10.1145/3411764.3445211

[CR26] Marres N (2018). Why we can’t have our facts back. Engag Sci Technol Soc.

[CR27] Marwick AE (2018). Why do people share fake news? A sociotechnical model of media effects. Georget Law Technol Rev.

[CR28] McAboy K (2020) Woman refusing to wear face covering at grocery store caught on video. Fox10Phoenix. Available at https://www.fox10phoenix.com/news/woman-refusing-to-wear-face-covering-at-grocery-store-caught-on-video. Accessed 01 June 2020

[CR29] Peck R (2018). Fox populism: branding conservatism as working class.

[CR30] Polletta F, Callahan J (2017). Deep stories, nostalgia narratives, and fake news: storytelling in the trump era. Am J Cult Sociol.

[CR31] Roberts ST (2019). Behind the screen: content moderation in the shadows of social media.

[CR310] Sauer MA, Truelove S, Gerste AK, Limaye RJ (2021) A Failure to Communicate? How Public Messaging Has Strained the COVID-19 Response in the United States. Health Security 19(1):65–74 10.1089/hs.2020.0190 PMC9195491 33606575

[CR100] Shabad R (2021) Rep. Marjorie Taylor Greene refers to Nazi-era 'brown shirts' in opposing vaccination push. NBC News. https://www.nbcnews.com/politics/congress/rep-marjorie-taylor-greene-refers-nazi-era-brown-shirts-opposingn1273204

[CR101] Shabad R, Ann Caldwell L, Talbot H (2021) Democrats shelve censure resolution after Rep. Greene apologizes for Holocaust comparison. NBC News. https://www.nbcnews.com/politics/congress/rep-marjorie-taylor-greene-apologizes-comparing-mask-wearingholocaust-after-n1270894

[CR32] Singh VK, Ghosh I, Sonagara D (2020). Detecting fake news stories via multimodal analysis. J Assoc Inform Sci Technol.

[CR33] Small ML (2009). How many casese do I need?: On science and the logic of case selection in field-based research. Ethnography.

[CR34] Swearer A (2020) The utterly American history of ‘We Will Not Comply” The Heritage Foundation. Available at https://www.argusobserver.com/opinion/the-utterly-american-history-of-we-will-not-comply/article_4cd0128e-4cf5-11ea-8aeb-ebdee3b88199.html. Accessed 11 Feb 2020

[CR35] Tacchini E et al (2017) Some like it hoax: automated fake news detection in social networks. Available at https://arxiv.org/abs/1704.07506. Accessed 01 Jan 2021

[CR37] Tripodi F (2018) Searching for alternative facts. Data & Society Research Institute. Available at https://datasociety.net/wp-content/uploads/2018/05/Data_Society_Searching-for-Alternative-Facts.pdf. Accessed 02 Jan 2021

[CR38] Trist EL, Bamforth KW (1951). Some social and psychological consequences of the longwall method of coal-getting. Hum Relat.

[CR39] Uyheng J, Carley KM (2020). Bots and online hate during COVID pandemic: case studies in the United States and the Philippines. J Comput Social Sci.

[CR40] Uyheng J, Bellutta D, Carley KM (2020) From xenophobia to political confrontation: shifting online discussions of racism during the COVID pandemic. In: Presented at the social-cybersecurity in times of crisis and change IDeaS Virtual Institute—November 19–20, 2020

[CR41] Wamsley L (2020) Fired Florida Data Scientist launches a coronavirus dashboard of her own. NPR. Available at https://www.npr.org/2020/06/14/876584284/fired-florida-data-scientist-launches-a-coronavirus-dashboard-of-her-own. Accessed 11 Dec 2020

[CR42] Wang T (2016) Why big data needs thick data. In: Ethnography matters. Available at https://medium.com/ethnography-matters/why-big-data-needs-thick-data-b4b3e75e3d7. Accessed 06 Jan 2021

